# Virome of the fungi associated with mushroom dry bubble disease

**DOI:** 10.1016/j.virusres.2026.199714

**Published:** 2026-03-18

**Authors:** Lóránt Hatvani, Sakae Hisano, Hideki Kondo, Hitomi Sugahara, Paul Telengech, Sabitree Shahi, Sarah Remi Ibiang, Sándor Kocsubé, Tünde Kartali, David A. Fitzpatrick, Helen Grogan, Nobuhiro Suzuki

**Affiliations:** a Teagasc Food Research Center, Horticulture Development Department, Ashtown, Dublin 15, D15KN3K, Ireland; b Institute of Plant Science and Resources, Okayama University, Chuou 2-20-1, Kurashiki, Okayama 710-0046, Japan; c Department of Biotechnology and Microbiology, Faculty of Science and Informatics, University of Szeged, Szeged, Közép fasor 52., H-6726, Hungary; d Genome Evolution Laboratory, Department of Biology, Maynooth University, Maynooth, Co. Kildare, Ireland; e Neovirology Laboratory, Graduate School of Agricultural Science, Tohoku University, Sendai, Japan

**Keywords:** Lecanicillium fungicola, Agaricus bisporus, Akanthomyces, Simplicillium, dsRNA, Myovirus, Fungal virus, Mycovirgaviridae, Partitiviridae, Polymycoviridae, Botourmiaviridae, Splipalmiviridae, Narna-like virus

## Abstract

Dry bubble disease, attributed to the filamentous fungus *Lecanicillium fungicola* (Cordycipitaceae) results in huge yield losses in mushroom (*Agaricus bisporus*) cultivation worldwide. The possibilities for controlling the disease using commercial fungicides are highly limited, and therefore, there is an increasing demand for novel, alternative means of pest management. Our research objective was the comprehensive examination of viruses in the causal agents of dry bubble disease, which may open up an avenue for its virocontrol in the future. Out of 57 fungal isolates obtained from dry bubble-affected *A. bisporus* crops in various countries, 47 (82%) were confirmed by ITS (Internal Transcribed Spacer) sequence analysis as *L. fungicola*. In addition, different members of the genera *Akanthomyces* and *Simplicillium* (7 and 3 isolates, respectively), yet unknown to cause dry bubble symptoms, have also been detected. Cellulose column chromatography revealed the presence of double-stranded (ds) RNA in seven *L. fungicola* and three *Akanthomyces* sp. isolates, suggesting viral infection. The ten dsRNA-positive and eight randomly selected dsRNA-negative fungal strains were subjected to rRNA-depletion high-throughput RNA-sequencing analysis. The presence of seven new viruses representing four new species in the established families, *Partitiviridae, Polymycoviridae, Botourmiaviridae* and the narna-like virus group, and three previously established/proposed species in the families *Chrysoviridae* and “*Mycovirgaviridae*” were confirmed. The impact of the detected and identified viruses on their host fungi, and their potential applicability for virocontrol purposes will be examined in the future. This study provides the first detailed report on viruses of mushroom pathogenic fungi.

## Highlights

• Mushroom (Agaricus bisporus)-pathogenic fungi were screened for virus infections.
• Tested fungivorous fungi included Lecanicillium, Simplicillium, and Akanthomyces spp.
• Seven novel RNA virus strains were fully sequenced.
• Four represent novel species while three belong to previously reported species.
• This provides the first detailed report on viruses of mushroom-pathogenic fungi.

## 1 Introduction

World production of cultivated edible mushrooms continues to grow and was worth US$ 42 billion in 2018, compared to US$ 34 billion in 2013, with *Lentinula, Pleurotus, Auricularia, Agaricus* and *Flammulina* species accounting for 85% of total production (Royse et al., 2017). In Europe, button mushroom (*Agaricus bisporus*) is predominant (Royse et al., 2017; Singh et al., 2020). As in other agricultural crops, several pests and pathogens - such as viruses, bacteria, fungi, nematodes, arthropods - can affect mushroom cultivation, resulting in significant crop losses and, consequently economic problems (Grogan, 2008). The important fungal pathogens of cultivated mushrooms include fungivorous fungal species of *Trichoderma, Cladobotryum, Mycogone* and *Lecanicillium*, which cause green mould, cobweb, wet bubble and dry bubble disease, respectively, with a continuously widening spectrum (Gea et al., 2011; Hatvani et al., 2017; Potocnik et al., 2015). Although certain chemical fungicides are used to control these fungi, intrinsic or acquired resistance of the pathogens (Gea et al., 2005; Grogan, 2006) narrows their applicability. Integrated pest management, involving biological tools, has been suggested as a potential solution for disease control to safeguard human health as well as the environment by reducing unnecessary pesticide use (Grogan, 2008). There are successful examples of practical or experimental biological control of destructive phytopathogenic fungi causing serious damages in crops, including biocontrol of the chestnut blight fungus (*Cryphonectria parasitica*) using a hypovirulece-inducing single-stranded (ss) RNA hypovirus (Nuss, 1992; Rigling and Prospero, 2018), and that of rapeseed stem rot (caused by *Sclerotinia sclerotiorum*) using a DNA virus (Xie and Jiang, 2024; Zhang et al., 2020). These inspired us to investigate the understudied virome of *L. fungicola*, the causal agent of dry bubble, one of the most destructive diseases in the commercial production of the white button mushroom (Berendsen et al., 2010), in the hope that viruses with potential to serve as a biological control agent could be found.

Fungal virome studies would greatly help find biocontrol agents against mushroom pathogenic fungi and simultaneously contribute to a better understanding of virus diversity and evolutions (Kondo et al., 2022). In addition to the above-mentioned fungal viruses infecting *C. parasitica* and *S. sclerotiorum*, mycovirus candidates for biocontrol agents have been identified from various basidiomycetes (e.g., (Ayllon and Vainio, 2023; Vainio and Hantula, 2016) and ascomycetes (Kondo et al., 2013; Li et al., 2019; Xie and Jiang, 2014; Yu and Kim, 2020), although there is a huge gap between identification of potential biocontrol agents at the laboratory level and successful biocontrol practice using them in the field. Virus hunting studies of basidiomycetes and ascomycetes revealed a unique group of viruses (Forgia et al., 2021; Sutela et al., 2020), now classified in the new phylum *Ambiviricota* (Kuhn et al., 2024), members of which show intermediate nature between viroids and RNA viruses. Ambiviruses have a circular RNA genome encoding both ribozymes and RNA-dependent RNA polymerase (RdRP). Other examples include the discovery of splipalmiviruses with a multisegmented positive sense (+) RNA genome using a split RdRP coding strategy and RNA viruses with new lifestyles (Sato et al., 2023a) as exemplified the yadokari/yadonushi nature (Hisano et al., 2018) and hadaka nature (Sato et al., 2023b). These findings have been achieved largely by screening large collections of phytopathogenic fungi and oomycetes, while several virome studies with entomopathogenic (Herrero et al., 2012; Kotta-Loizou and Coutts, 2017b) and human pathogenic fungi (Ejmal et al., 2018; Jiang et al., 2019; Kanhayuwa et al., 2015; Kotta-Loizou and Coutts, 2017a), and non-pathogenic fungi (Myers et al., 2020) also contributed. Previous studies have reported the virome of ascomycetes pathogenic to insects and taxonomically related to the above-mentioned mushroom pathogens, i.e., *Beauveria bassiana* (Herrero et al., 2012; Kotta-Loizou and Coutts, 2017b; Shi et al., 2024) and *Metarhizium* spp. (Guo et al., 2024). As far as we are aware, there are no or few reports on viruses infecting the above-mentioned mushroom-pathogenic fungi except for *Trichoderma* spp. and *Mycogone perniciosa* (e.g., Fletcher et al., 1995; Yun et al., 2016).

In this study, we screened a total of 57 fungal isolates, mostly associated with dry bubble disease, for virus infections by conventional dsRNA assay and subsequently RNA-Seq analysis. This study reveals the presence of new virus strains belonging to four species and three previously established or proposed species.

## 2 Materials and methods

### 2.1 Fungal strains and culture conditions

A total of fifty-seven fungal strains were collected largely from dry bubble-affected mushroom crops. Provenances of the examined fungal strains is described in Table S1. The examined fungal cultures were identified based on their internal transcribed spacer (ITS) sequences (Table S1, Fig. S1). The tested fungi included 47 isolates of *L. fungicola*, seven isolates of *Akanthomyces* spp., and three isolates of *Simplicillium* spp*.*, originating largely from diseased mushroom crops. Interestingly, on top of *L. fungicola*, additional species belonging to the family *Cordycipitaceae*, including *Simplicillium* spp. and *Akanthomyces* spp., previously unknown to be associated with dry bubble disease, were also identified (Table S1, Fig. S1). The cultures were preserved in 50% (v/v) glycerol at −80 °C, and unless specified, all cultivations were carried out on Potato Dextrose Agar (PDA, Difco™) medium at 25 °C.

### 2.2 RNA preparation

Total RNA and dsRNA fractions were prepared as described by Eusebio-Cope and Suzuki (Eusebio-Cope and Suzuki, 2015). Briefly, fungal mycelia were homogenized using mortar and pestle with liquid nitrogen. The mycelial powder was subjected to extraction with phenol-chloroform and two rounds of chloroform purification, followed by ethanol precipitation and treatment with RQ1 RNase free DNase I (Promega). After being deproteinized with phenol-chloroform, total RNA fractions were ethanol-precipitated.

DsRNA was isolated using cellulose powder (cellulose powder B3; Advantec Co., Ltd., Tokyo, Japan) after extraction with phenol:chloroform:isoamyl-alcohol. The dsRNA fractions were further treated doubly by RQ1 RNase free DNase I (Promega) and S1 Nuclease (Takara Bio Inc., Kusatsu, Shiga, Japan).

The obtained RNA fractions were examined by 1% (w/v) agarose gel electrophorese in the 0.5X TAE buffer system (20 mM Tris-acetate, 0.5 mM EDTA, pH 7.8) and spectrometrically using NanoDrop One (Thirmo Scientific, Waltham, MA, USA).

### 2.3 Sequence and phylogenetic analyses

**Fig. 1:**
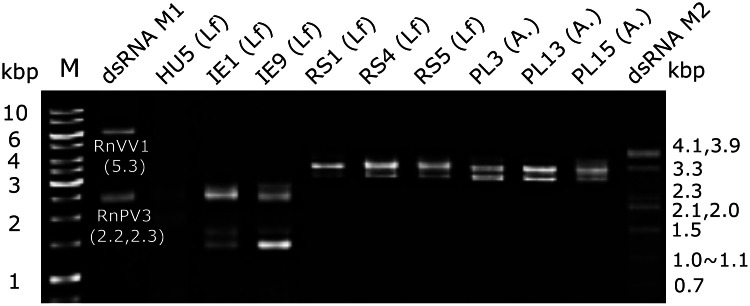
**DsRNA profiles of dsRNA-positive fungal strains.** A dsRNA fraction was obtained from each of nine fungal strains previously tested dsRNA-positive (Table S1) by a conventional method (see Materials and Methods). Tested fungal strains *Lecanicillium fungicola* (Lf) and *Akanthomyces* sp. (A.) include: HU5, IE1, IE9, RS1, RS4, RS5, PL3, PL13, and PL15. Note that the fungal strain R2, which originally tested dsRNA-positive, lost dsRNA elements during storage in the laboratory. Mycelia were harvested from two PDA-cellophane plates to purify dsRNA. Obtained dsRNA fractions were suspended in distilled water with final concentration adjusted to 200 ng/μl. One μl of the purified dsRNA solution was applied to a well of 1.0% (w/v) agarose gel and run in the 0.5 × TAE (20 mM Tris-acetate, 0.5 mM EDTA, pH 7.8) buffer system. The size standards used are the 1 kb dsDNA ladder size marker (GeneRuler 1 kb DNA ladder, Thermo Fisher) and dsRNA markers: Rosellinia necatrix victorivirus 1 (RnVV1, 5.3 kbp, AB742454) + Rosellinia necatrix partitivirus 3 (RnPV3, 2.2 + 2.3 kbp, LC010950, LC10951) (lane dsRNA M1) and mycoreovirus 1 (MyRV1) genomic dsRNA of 0.7 kbp∼4.1 kbp (lane dsRNA M2) (Suzuki et al., 2004).

Total RNA samples obtained from ten dsRNA-positive fungal strains of *L. fungicola* (IE1, IE9, RS1, RS2, RS4, RS5 and HU5) and *Akanthomyces* spp. (PL3, PL13, PL15) were analysed individually by high-throughput sequencing (HTS) (Fig. 1 and data not shown). Additionally, total RNA samples from eight randomly selected dsRNA-negative fungal strains of L. *fungicola* (IE14, UK2, CBS 440.34, NBRC 30728, MAFF 305218, and TUFC 65020 and DC114) and *S. lamellicola* (PL14) were also subjected to HTS analysis. See Table S1 for fungal strains included in HTS analysis. The total RNA samples were sent to Genome Lead Co. (Takamatsu, Kagawa, Japan), where 150 base pair (bp) paired-end sequencing using an MGI DNBSEQ G400RS was conducted after ribosomal RNA depletion (QIAseq FastSelect-rRNA Yeast kit, Qiagen, Hilden, Germany) and library preparation (MGIEasy RNA Directional Library Prep Set, MGI Japan, Tokyo, Japan). After quality checking and adapter trimming, raw sequence reads were assembled *de novo* using the CLC Genomics Workbench with default parameters (length fraction = 0.5; similarity fraction = 0.8) (version 20, CLC Bio-Qiagen, Aarhus, Denmark), as described by Kondo et al. (Kondo et al., 2025). A Basic Local Alignment Search Tool (BLAST) X database search (Altschul et al., 1997) was run to compare the contigs to viral reference sequences (Release 229) from the National Center for Biotechnology Information (NCBI). In addition, although we utilized a newer viral RdRP database, NeoRdRp2 (Sakaguchi et al., 2024), no additional virus-like sequence contigs were detected.

Phylogenetic analyses of newly discovered viruses were conducted according to Kondo et al. (Kondo et al., 2023) with slight modifications, based on sequence alignment of the RdRP region, using MAFFT online ver. 7 (Katoh and Standley, 2013). Poorly aligned sites were removed using trimAI version 1.41 on the NGPhylogeny.fr web service (Capella-Gutierrez et al., 2009; Lemoine et al., 2019) (https://ngphylogeny.fr/). Phylogenetic trees were generated using the IQ-TREE web server and supported by SH-aLRT/ultrafast bootstrap analysis (Trifinopoulos et al., 2016) (https://iqtree.github.io). The best-fit model was chosen according to the BIC criterion using ModelFinder (Kalyaanamoorthy et al., 2017), which is implemented in the Q-TREE web server. The trees were visualized using FigTree ver. 1.3.1 software (https://tree.bio.ed.ac.uk/software/figtree/).

### 2.4 RACE analyses

Two approaches were taken to determine the terminal sequences of detected viruses. One was the conventional 5′ RNA ligase mediated rapid amplification of cDNA ends (RLM-RACE) and Sanger sequencing as previously described (Fadli et al., 2025). Approximately 3 μg of total ssRNA (botourmiavirus and splipalmivirus) or dsRNA fractions (all dsRNA viruses and mycovirgavirus) was denatured in 90% (v/v) DMSO and subjected to RLM-RACE adaptor ligation (5′-PO_4__—_CAATACCTTCTGACCATGCAGTGACAGTCAGCATG-3′) with T4 RNA ligase (TaKaRa, Kusatsu, Japan) and RNase inhibitor (Toyobo, Osaka, Japan). cDNA was synthesized on the ligate with M-MLV reverse transcriptase (Promega) and used as template in PCR. The primers used in the cDNA synthesis and PCR amplification are listed in Table S2. The other was circular RACE and Sanger sequencing, which was applied for the splipalmivirus, chrysovirus, mycovirgavirus, and botourmiavirus. Generally, circular RACE of mRNA entails a decapping step with tobacco pyrophosphatase and a circularization step with T4 RNA ligase (McGrath, 2011). However, in this study, circular RACE was performed without these steps, in which total RNA was used as template. The consensus sequence of each terminus was obtained based on the alignment of at least five independently sequenced clones using GENETYX®-MAC Network Ver. 21 software (GENETYX Corp., Tokyo, Japan).

### 2.5 Confirmation of fungal strains hosting viruses by RT-PCR

Many viral contigs were detected by HTS from the RNA pools. To identify host fungal strains, one-Step RT-PCR was performed using the Takara One-Step RT-PCR Kit (Takara Bio) (Sato et al., 2020b; Urayama et al., 2015). Total RNA fractions were prepared as above from mycelia grown on PDA plates overlaid with cellophane membranes (cat. #. 89196457**;** pore size PT#300; 30 g/m², Heiko Pack, Ltd., Haga, Tochigi, Japan), and used as RNA substrate. Primer pairs were designed based on the contig sequences (Table S2).

## 3 Results and discussion

All fungal strains listed in Table S1 were tested for presence of dsRNA. A total of ten dsRNA-positive strains (IE1, IE9, HU5, RS1, RS2, RS4, RS5, PL3, PL13, and PL15) were subjected for HTS analyses using separate RNA materials, as described in Materials and Methods. DsRNA profiles of some dsRNA-positive strains are mostly shown in Fig. 1. Eight dsRNA-negative strains (IE14, PL14, UK2, CBS 440.34, DC 114, NBRC 30728, MAFF 305218, and TUFC 65020) were also examined by HTS. All detected viruses, which were fully sequenced in this study, are summarized in Table 1. These partial genomic sequences were used to design primers for RLM-RACE or circular RT-PCR for determining their entire genomic sequences. First, we confirmed which fungal strain hosts the virus predicted from HTS data by RT-PCR (data not shown). We successfully determined the entire genomic sequences of eight virus strains (Table 1). To determine if the characterized viruses represent new species, ICTV demarcation criteria for established genus members was implemented <https://ictv.global/>. When viruses of interest belong to as-yet-unestablished families or genera (mycovirgaviruses), 70% RdRP amino acid (aa) sequence identity was tentatively set as a species demarcation criterion in this paper.

**Table 1:** List of completely sequenced virus genomes.

Virus name (abbreviation)	Host fungal strain	Contig No	Genomic segment	Length (nt or bp)	Accession no	BlastP top hit	Query Coverage (%)	Identity (%)
Lecanicillium fungicola partitivirus 1 (LfPV1)	*Lecanicillium fungicola* var. *fungicola* HU5	2	dsRNA1	1766	LC912012	Beauveria bassiana partitivirus 1	92	91.65
		4	dsRNA2	1632	LC912013	Beauveria bassiana partitivirus 1	81	85.68
Lecanicillium fungicola polymycovirus 1 (LfPmV1)	*Lecanicillium fungicola* var*. fungicola* HU5	6	dsRNA1	2422	LC912014	Cladosporium cladosporioides virus 1	94	65.09
		8	dsRNA2	2206	LC912015	Cladosporium cladosporioides virus 1	92	59.71
		23	dsRNA3	2064	LC912016	Setosphaeria turcica polymycovirus 2	91	60.13
		7	dsRNA4	1181	LC912017	Cladosporium cladosporioides virus 1	71	51.59
Akanthomyces sp chrysovirus 1 (AsCV1)	*Akanthomyces* sp. PL15	38	dsRNA1	3510	LC912018	Beauveria bassiana chrysovirus 1	100	88.25
		17	dsRNA2	3187	LC912019	Beauveria bassiana chrysovirus 1	90	76.21
		13	dsRNA3	3022	LC912020	Beauveria bassiana chrysovirus 1	90	66.38
		5	dsRNA4	2788	LC912021	Beauveria bassiana chrysovirus 1	92	78.31
Lecanicillium fungicola chrysovirus 1 (LfCV1)	*Lecanicillium fungicola* var. *fungicola* RS5	9	dsRNA1	3559	LC912022	Beauveria bassiana chrysovirus 1	93	83.47
		10	dsRNA2	3238	LC912023	Beauveria bassiana chrysovirus 1	86	56.91
		7	dsRNA3	3104	LC912024	Beauveria bassiana chrysovirus 1	91	63.99
		4	dsRNA4	2817	LC912025	Beauveria bassiana chrysovirus 1	90	72.74
Lecanicillium fungicola mycovirgavirus 1 (LfMvV1)	*Lecanicillium fungicola* var. *fungicola* RS5	1	RNA1	1806	LC912026	Erysiphe necator associated abispo virus 2	78	73.32
		2+60	RNA2	1780	LC912027	Penicillium glabrum RNA virus 1*	90	98.88
		6	RNA3	891	LC912028	No hit		
		3	RNA4	839	LC912029	No hit		
Lecanicillium fungicola narna-like virus 1 (LfNLV1)	*Lecanicillium fungicola* var*. fungicola* NBRC 30728	102	RNA1	1873	LC912030	Rhopalosiphum padi narna-like virus 1*	100	83.79
		212	RNA2	1660	LC912031	Streptophyte associated narna-like virus 19*	95	67.28
		319	RNA3	1277	LC912032	No hit		
Simplicillium lamellicola botourmiavirus 1 (SlBoV1)	*Simplicillium lamellicola* PL14	55	undivided	2198	LC912033	Botourmiaviridae sp.*	85	67.14

⁎ : metagenome assembled genome (MAG) sequence.

### 3.1 dsRNA viruses

**Fig. 2:**
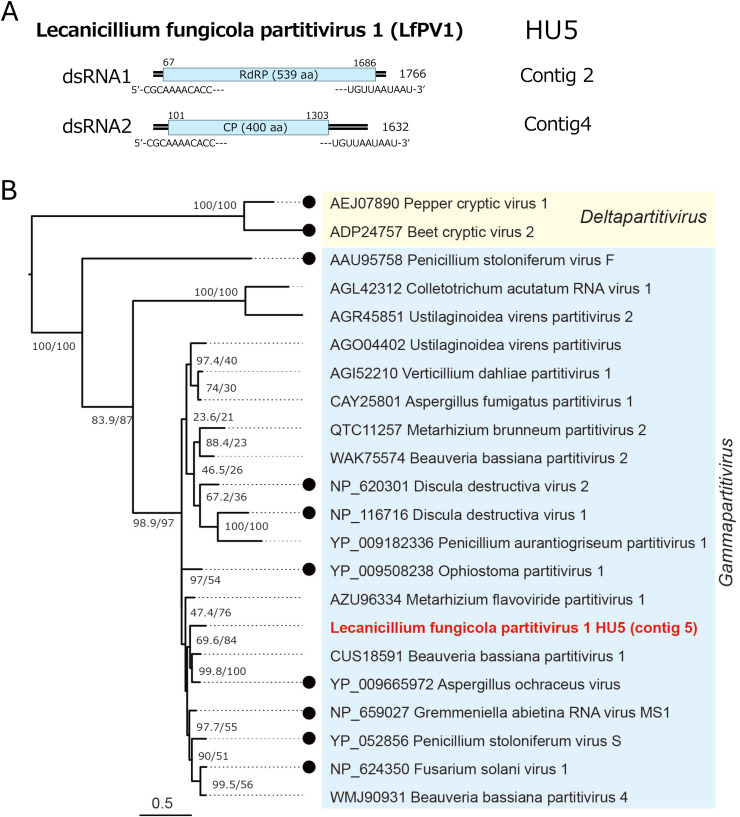
**Genome organization and phylogeny of Lecanicillium fungicola partitivirus 1 (LfPV1).** (A) Schematic diagram of the genome of LfPV1. The two genomic dsRNA (contigs 2 and 4) are 1766 bp and 1632 bp in length and each have the terminal conserved sequences 5′-CGCAAAACACC—-UGUUAAUAAU-3′ on their plus strands. Boxes indicate open reading frames (ORFs) with their coding capacity. LfPV1 was detectable in fungal strain HU5 (Table 1). (B) Phylogenetic relation of LfPV1 and gammapartitiviruses. The maximum likelihood (ML) tree was constructed based on the MAFFT alignment of gammapartitivirus RdRP sequences, using the LG+*I* + G4 model as the best-fit substitution model. Two deltapartitiviruses were used as outgroups. In this and subsequent ML trees, virus names are preceded by their respective accession numbers, and members of established viral species are indicated by filled circles. Mycoviruses newly identified in this study are shown in bold with red letters. Clades within the tree are colored according to viral genera or related groups. The tree was visualized using FigTree ver. 1.3.1, and the scale bar represents amino acid substitutions per site. Numbers at the nodes indicate SH-aLRT/ultrafast bootstrap support values.

Five dsRNA viruses were isolated from four fungal strains: HU5, IE9, PL15, and RS5. Strain HU5 harboured a partitivirus termed Lecanicillium fungicola partitivirus 1 (LfPV1) with properties typical of a gammapartitivirus (family *Partitiviridae*, order *Durnavirales*). The genomic segments sizes of LfPV1 are 1766 bp for dsRNA1 and 1632 bp for dsRNA2 with the conserved terminal sequences (5′-CGCAAAACACC—–UGUUAAUAAU-3′) (Fig. 2A). The 5′-CGCAAAA—is conserved in many gammapartitiviruses (Nibert et al., 2014; Vainio et al., 2018). Homology search showed LfPV1 dsRNA1 and dsRNA2 to encode RdRP of 539 aa and capsid protein (CP) of 400 aa, respectively, with sequence similarity to counterparts of gammapartitiviruses. While LfPV1 showed a high phylogenetic affinity to Beauveria bassiana partitivirus 1 (Kotta-Loizou and Coutts, 2017b) (Fig. 2B) with high levels of RdRP and CP sequence identity over 80% but below 90% (Table 1), it belongs to a new species according to the current species demarcation criteria: <90% identity for RdRP and <80% identity for CP (Vainio et al., 2018).

The genome of two alphachrysoviruses (family *Chrysoviridae*, order *Ghabrivirales*) from two fungal strains *Akanthomyces* sp. PL15 and *L. fungicola* RS5, which were designated as Akanthomyces sp chrysovirus 1 (AsCV1) and Lecanicillium fungicola chrysovirus 1 (LfCV1), were fully determined. These two viruses show 83.4% RdRP sequence identity. A BLASTP search showed both viruses to share the highest RdRP amino acid sequence identities (>80%) to Beauveria bassiana chrysovirus 1 (BbCV1) (Gilbert et al., 2019). Pairwise comparison revealed a close relationship among the two newly identified chrysoviruses and BbCV1, which exhibited over 80% RdRP sequence identity, thus all belonging to the same previously established species *Alphachrysovirus isariae*. The LfCV1 genome comprises four dsRNA segments (dsRNA1 to dsRNA4), each possessing a large single ORF (Fig. S1A). The genomic segments each encode RdRP of 1113 aa (dsRNA1), CP (dsRNA2), P3 (dsRNA3) and P4 (dsRNA4). As for other chrysoviruses (Jiang and Ghabrial, 2004; Shahi et al., 2021; Urayama et al., 2010), both the terminal sequences (5′-AUAAAACAAAA——-GGUUUUAAAAGCG-3′ on the plus strand) are shared among the four genomic segments. It is noteworthy that the terminal sequences of AsCV1 (5′-AUAAAACAAAAUCC——-AAAGCG-3′ on the plus strand) are nearly identical to those of LfCV1 (Fig. S1B). While mini-cistrons preceding the large ORFs are occasionally observed in alphachrysoviruses (Kotta-Loizou et al., 2020; Shahi et al., 2021), no such mini-cistron was observed in any segment of the two chrysoviruses: AsCV1 and LfCV1. Notable recent findings with chrysoviruses other than the presence of small ORFs preceding large ORFs include the internal ribosome entry site (IRES) activity of 5′ untranslated region of chrysovirus mRNAs (Chiba et al., 2018), and identification of ribozymes (López-Galiano et al., 2024), identification of the nucleotide governing both activities of the IRES and ribozyme. Interestingly these characteristics are well documented in alphachrysoviruses but not in betachrysoviruses. The alphachrysovirus discovered in this study possesses most of the above-mentioned properties.

Other chrysovirues with nearly 100% RdRP sequence identity to AsCV1 PL15 or LfCV1 RS5 were detected from fungal strains PL3 and PL13, or RS1, RS2, and RS4, respectively (Table S1). It is of interest to note that the two viruses were commonly originated from either of the two countries: Serbia and Poland. LfCV1 and AsCV1 phylogenetically form a clade along with other alphachrysoviruses (Fig. S1B).

**Fig. 3:**
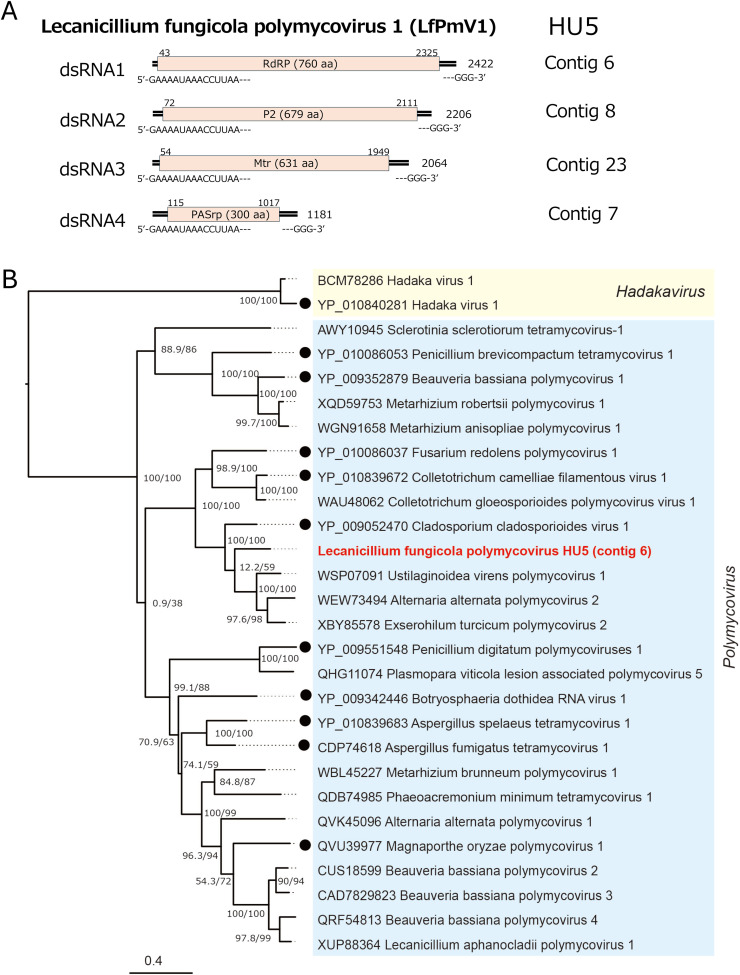
**Genome organization and phylogeny of Lecanicillium fungicola polymycovirus 1 (LfPmV1).** (A) Schematic diagram of the LfPmV1 genome organization. The four genomic dsRNA segments (dsRNA1 to dsRNA4) each have single ORFs that would encode P1 (RNA-dependent RNA polymerase), P2 (hypothetical protein with unknown function), P3 (methyltransferase), or P4 (proline-alanine‑serine rich protein, PASrp), respectively. Black boxes indicate ORFs with their coding capacity. The coding strands of the four genomic segments share the terminally conserved sequence stretches, 5′-GAAAAUAAACCUUAA—GGG3'. (B) Phylogenetic analysis of LfPmV1. The ML tree was constructed based on the MAFFT alignment of polymycovirus RdRP sequences, using the LG+*F* + *I* + G4 model as the best-fit substitution model. Two strains of a hadakavirus were used as outgroups.

A polymycovirus, termed Lecanicillium fungicola polymycovirus 1 (LfPmV1), was detected from three fungal strains (HU5, IE9 and IE1) of *L. fungicola*. Two polymycovirus isolates from fungal strains HU5 and IE) were completely sequenced, while the complete coding regions of the IE9 isolate of LfPmV1 was obtained from HTS. All of these polymycoviruses, belonging to an orphan family *Polymycoviridae*, have four genomic segments encoding three core proteins: RdRP (domain cd01699) (dsRNA1), S-adenosylmethionine-dependent methyltransferases (AdoMet_MTases, domain cd02440) methyltransferase (Mtr, dsRNA3), and proline-alanine‑serine-rich protein" (PASrp, dsRNA4) and a hypothetical protein (dsRNA2) (Fig. 3A). LfPmV1 dsRNA1 to dsRNA4 shared the terminally conserved sequences (5′-GAAAAUAAACCUUAA—GGG3-’). The three polymycoviruses have properties expected for polymycoviruses. A pairwise comparison showed a high level (>97.7%) of RdRP amino acid sequences among the polymycoviruses from HU5, IE9, and IE1, suggesting that these polymycoviruses belong to the same species. The LfPmV1 RdRPs shows the highest sequence identity (approximately 65%, 100% coverage) by BLASTP to that of the closest relative, Cladosporium cladosporioides virus 1 (accession no YP_009052470). Thus, the polymycoviruses detected from *L. fungicola* all belong to a new species, as the species demarcation criteria for the polymycoviruses are below 70% identity in RdRP sequences (Kotta-Loizou et al., 2022). This is supported from an RdRP-based phylogenetic tree (Fig. 3B).

The segment numbers of polymycoviruses reported thus far range from 4 to 8 and they encode three core proteins (Kotta-Loizou et al., 2022), while three hallmark genes are observed in all polymycoviruses: genes encoding RdRP, Mtr, and PASrp. Two morphological entities were reported for different polymycoviruses: one is PASrp associated dsRNA-protein complex (Kanhayuwa et al., 2015) and filamentous particles composed of dsRNA and PASrp (Han et al., 2025; Jia et al., 2017). The dsRNA in the former type is inaccessible by exogenous RNase (Sato et al., 2020a). The polymycovirus dsRNA, whether associated with PASrp or naked, was shown to be infectious (Han et al., 2025; Jia et al., 2017; Kanhayuwa et al., 2015), similar to the replicated form dsRNA of a yadokarivirus (the phylum *Pisuviricota*) from *Aspergillus foetidus* (Fadli et al., 2025). These observations led the ICTV to classify polymycoviruses as dsRNA viruses (Kotta-Loizou et al., 2022), even though they show close phylogenetic affinity to hadakaviruses (family *Hadakaviridae*) in the phylum *Pisuviricota* with (+)RNA genomes (Sato et al., 2023b). Despite these interesting attributes of polymycoviruses, they have been understudied.

### 3.2 (+)RNA viruses

**Fig. 4:**
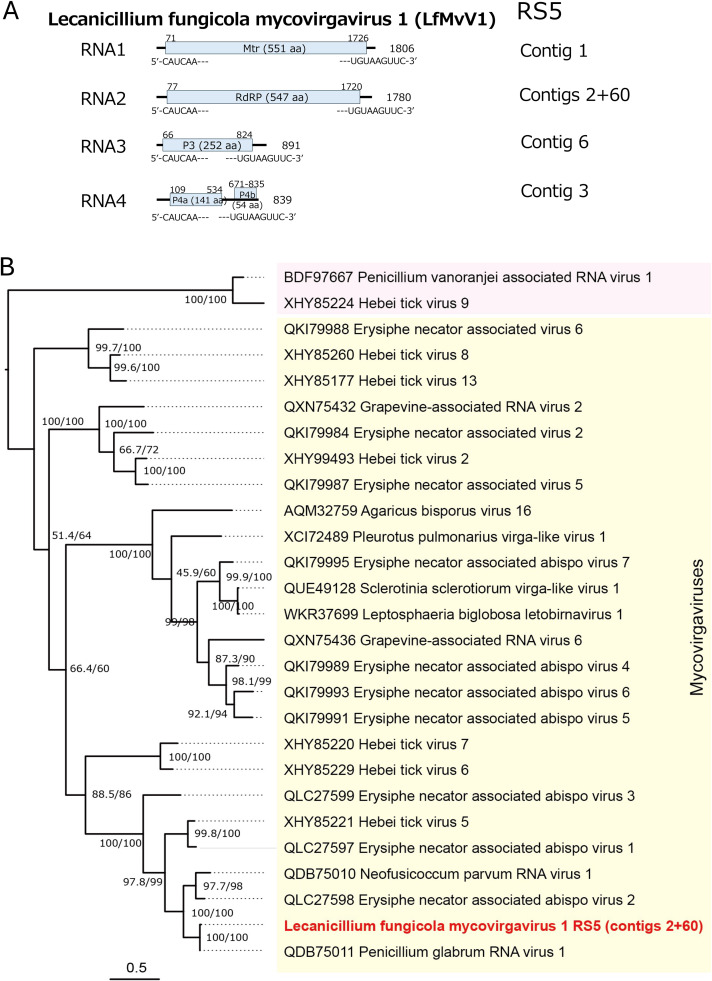
**Genome organization and phylogeny of Lecanicillium fungicola mycovirgavirus 1 (LfMvV1).** (A) Diagrammatic representation of the genome of the LfMvV1 genome organization. Each of the three genomic dsRNA segments (RNA1 to RNA3) possesses a single ORF that would encode P1 (methyltransferase), P2 (RdRP), or P3 (hypothetical protein with unknown function), respectively. The smallest genomic segment of LfMvV1 (RNA4) possesses two non-overlapping ORFs that would encode a 141-amino acid protein (P4a) and a 54-amino acid protein (P4b) with unknown function, respectively. The terminally sequence stretches, 5′-CAUCAA–UGUAAGUUC-3’ is strictly conserved across the coding strands of the four genomic segments. (B) Phylogenetic relationships of LfMvV1 and related viruses. A maximum likelihood tree was constructed based on a multiple alignment of the RdRP sequences from related viruses using the LG+ *I* + G4 model as the best-fit substitution model.

Three new (+)RNA virus strains were detected from three fungal strains: Lecanicillium fungicola mycovirgavirus 1 (LfMvV1), Simplicillium lamellicola botourmiavirus 1 (SlBoV1), and Lecanicillium fungicola narna-like virus 1 (LfNLV1). The first virus has a multi-segmented (+)RNA genome isolated from fungal strain RS5. Mycovirgaviruses also remain taxonomically unassigned by the ICTV, while non-segmented tobamo-like or virga-like viruses virus groups have recently been classified into the family *Tobaliviridae* (order *Martellivirales*) (Sabanadzovic et al., 2026). Virga-like mycoviruses likely with segmented genomes have been discovered as lately as splipalmiviruses (Deakin et al., 2017; Sutela et al., 2020). Single mycovirgavirus segments encoding RdRP were first discovered in *Penicillium glabrum* (Penicillium glabrum RNA virus 1, PgMvV1) and *Neofusicoccus parvum* (Neofusicoccum parvum RNA virus 1, NpMvV1) in 2019, which was proposed to be classified into a new family “Mycovirgaviridae” (Nerva et al., 2019). These viral segments exhibit high levels of amino acid sequence identity (98.9% or 73.0%) to the RdRP encoded by LfMvV1 RNA2 by BLASTP search. A mycovirga-like virus, Agaricus bisporus virus 16 (AbV16), was reported from mushroom (*Agaricus bisporus*), which appears to have four genomic RNA segments (RNA1 to RNA4) (Dobbs et al., 2021). The largest (RNA1) and second largest (RNA2) segments encode an Mtr and an RdRP, respectively, which share modest levels of sequence identity (27%∼32%) with the counterparts of LfMvV1. LfMvV1 RNA3 and RNA4 encode protein P3 (252 aa) and P4a (141 aa) of unknown function, for which no hit returned by BLASTP. Notably, RNA4 encodes an additional small protein P4b (54 aa) (Fig. 4A) with no aa sequence similarity to known proteins detectably by BLASTP search. Many other mycovirga-like viruses related to LfMvV1, including Erysiphe necator associated abispo virus 2 (GenBank accession no., QLC27598.1) and Sclerotinia sclerotiorum virga-like virus 1 (Jia et al., 2021), have been reported for which only segment sequences are available. LfMvV1 RNA1 to RNA4 share a strictly conserved terminal sequence stretch (5′-CAUCAA—-UGUAAGUUC-3′) (Fig. 4A). A phylogenetic tree generated based upon RdRP alignments cluster these mycovirgaviruses and myco-virga-like viruses together. We propose that PgMvV1, NpMvV1, and LfMvV1 belong to a same species within the “*Mycovirgavirdae*” (order *Martellivirales*). It shall be appropriate that multiple new genera or potentially new families should be created to accommodate AbV16 and other mycovirga-like viruses because of their distant relationship to mycovirgaviruses (Fig. 4B).

This study represents a molecular characterization of a mycovirgavirus (LfMvV1) with four genomic segments. However, mycovirgaviruses are still ill-explored and important key questions remain open. For example, how many core segments does a single mycovirgavirus have? Are rigid viral particles formed by mycovirgaviruses? If yes, what type of particles are formed? There should be further research to address these unanswered questions.

**Fig. 5:**
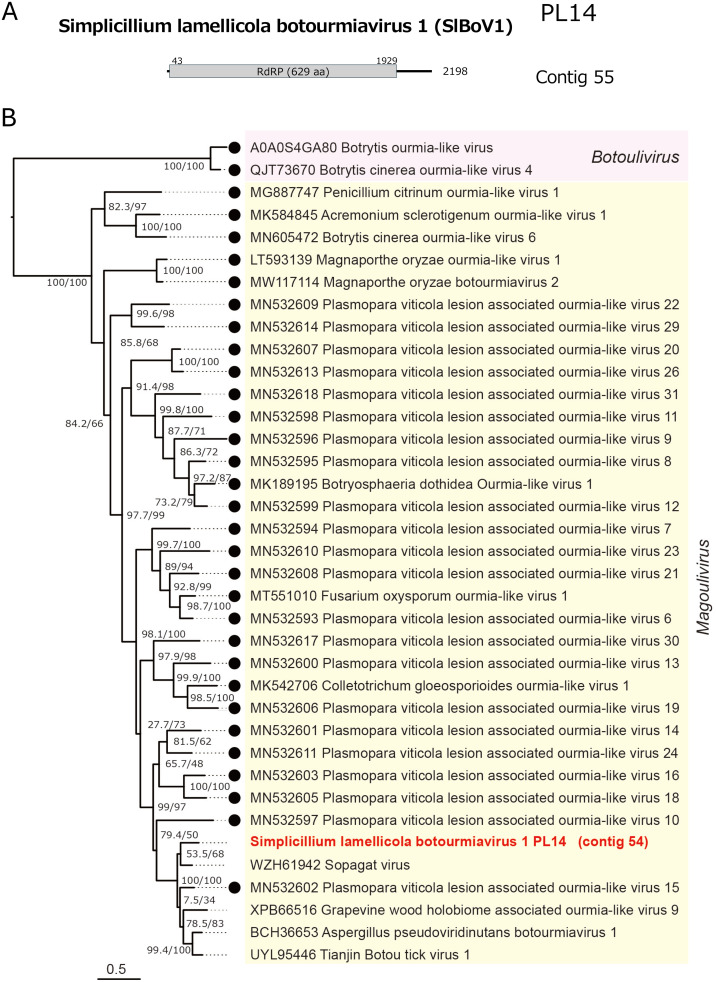
**Genome organization and phylogeny of Simplicillium lamellicola botourmiavirus 1 (SlBoV1).** (A) Genome organization of SlBoV1. The mono-segmented (+)RNA genome of SlBoV1 is 2198 nt in length and possesses a single ORF encoding RdRP alone. (B) Phylogenetic relationships of SlBoV1 and related magouliviruses. The ML tree was constructed based on a multiple alignment of the RdRP sequences from related viruses using the LG+*F* + *I* + G4 model as the best-fit substitution model. Two members of the genus *Botoulivirus* were used as outgroups.

The other two (+)RNA viruses were detected from two fungal strains (PL14 and NBRC 30728) (Table S1), which initially tested negative for dsRNA, suggesting the low-level accumulation of their replicative dsRNA. SlBoV1 has properties typical of a botourmiavirus in the genus within the family *Botourmiaviridae* (order *Ourlivirales*) (Ayllon et al., 2020): an RNA genome of 2198 nts in length with a single large ORF encoding RdRP (Fig. 5A). SlBoV1 RdRP shows high levels of sequence identity to previously reported botourmiaviruses such as Botourmiaviridae sp. (PMS18_Contig256 from *Erysiphe necator*), Suillus luteus botourmiavirus 3 (Liu et al., 2023), and Sopagat virus (Sadiq et al., 2024). According to the ICTV-established species demarcation criteria for botourmiaviruses (<90% RdRP aa sequence identity) (Ayllon et al., 2020), SlBoV1 belongs to a new species in the genus *Magoulivirus* (Fig. 5B).

**Fig. 6:**
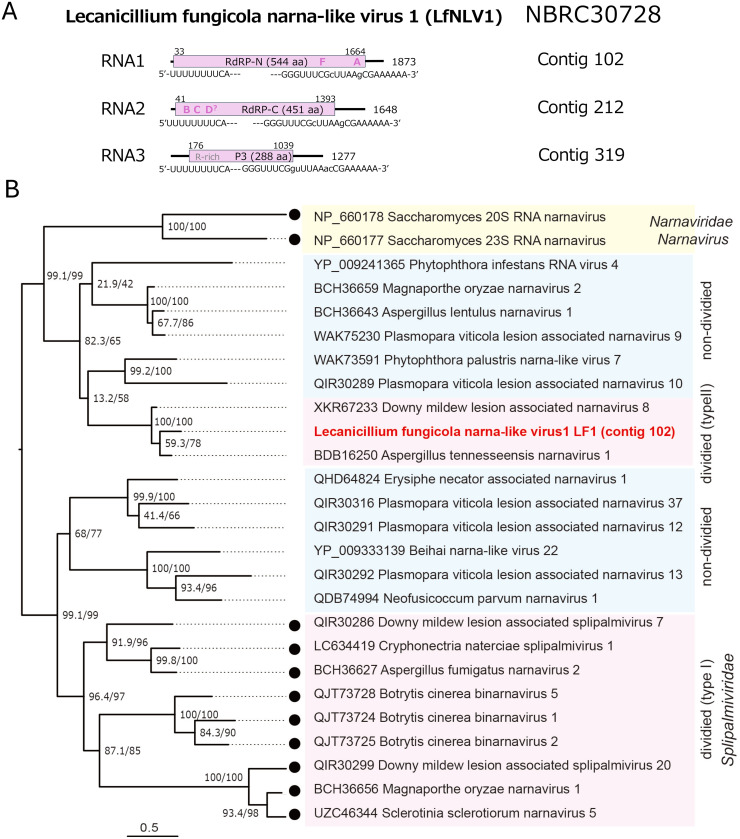
**Genome organization and phylogeny of Lecanicillium fungicola narna-like virus 1 (LfNLV1).** Genome organization of LfNLV1. LfNLV1 has three (+)RNA genomic segments that would encode the split RdRP domains (RNA1 and RNA2) and hypothetical protein (RNA3) homologous to those of other splipalmiviruses. The tri-segmented genome segments are 1873∼1277 nt in length with terminally shared sequences (5′-UUUUUUUUCA—- GGGUUUCGguUUAAacCGAAAAAA-3′). Note that lowercase letters in the parentheses refer to nucleotides that are less strictly conserved. (B) Phylogenetic relation of LfNLV1 and related viruses with divided and undivided RdRP domains. A midpoint rooting ML tree was constructed based on a multiple alignment of the RdRP sequences from related viruses within the order *Wolframvirales*. The LG+ *I* + G4 model was used as the best-fit substitution model.

Among many narna-like viruses (order *Wolframvirales*), several segmented RNA mycoviruses known as splipalmiviruses encode a unique, divided RdRP gene, which were first identified by a few research groups (Chiba et al., 2021a; Jia et al., 2021; Sato et al., 2022; Sutela et al., 2020). The family *Splipalmiviridae* was recently created officially by the ICTV (Sato et al., 2024) to accommodate many splipalmiviruses. In addition, viruses related to splipalmiviruses and narnaviruses (family *Narnaviridae*) have been reported largely from fungi and oomycetes, which have genomes encoding either divided or undivided RdRP domains. LfNLV1 exhibits a moderate level (approximately 70∼84%) of RdRP sequence identity to Rhopalosiphum padi narna-like virus 1, Zhangzhou Narna tick virus 4, Streptophyte associated narna-like virus 9 or 19 (SpANLV9, SpANLV19) (Feng et al., 2025; French et al., 2023). These virus sequences were discovered via metatranscriptomics analyses and are ill-explored biologically and molecularly. This study showed that LfNLV1 is likely to have 3 genomic segments, RNA1 to RNA3. The 5′ and 3′ terminal sequences are highly conserved between the segments (5′-UUUUUUUUCA—GGGUUUCGcUUAAgCGAAAAAA-3′), similarly to other splipalmiviruses (Fig. 6A). Note that lowercase letters in the parentheses refer to nucleotides that are less strictly conserved. LfNLV1 RNA1 and RNA2 encode the split RdRP domains, while RNA3 encode a protein homologous to counterparts of other splipalmiviruses (Table 1). Splipalmiviruses represent a group of peculiar viruses with the split palm domains of RdRP, hall mark of RNA viruses. Splipalmiviruses have been so far reported largely from ascomycetes with some from basidiomycetes (Chiba et al., 2021a; Chiba et al., 2021b; Daghino et al., 2024; De Miccolis Angelini et al., 2022; Sato et al., 2022). Plant pathogenic oomycetes such as *Bremia lactucae* (Forgia et al., 2024) and *Plasmopara viticola* are also implicated as their hosts (Chiapello et al., 2020), while its experimental validation is needed. Their genomic segment numbers appear to vary, ranging from 2 to 4, depending on viruses. A notable difference between LfNLV1 and previously reported splipalmiviruses is the split pattern for the RdRP domains; Motif B typified by RGVLMP is encoded by RNA2 upstream of motif C (GDD) in LfNLV1 unlike many splipalmiviruses encoding motif B by the RNA1 3′-terminal portion (Fig. 6A). Among several related viruses, two narna-like viruses, Aspergillus tennesseensis narnavirus 1 and downy mildew lesion associated narnavirus 8 (Chiapello et al., 2020; Chiba et al., 2021b), have a similar RdRP motif split pattern. This indicates variability in the division of splipalmivirus RdRP domains, and implies a different division event occurring on ancestors with undivided RdRP-encoding segments during the course of evolution. In this regard, it should be noted that some insect and plant RNA viruses have different orders of the RdRP domains with all encoded by single RNA species (Gorbalenya et al., 2002; Sabanadzovic et al., 2009). LfNLV1 may not belong to the family *Splipalmiviridae* based on our phylogenetic analyses (Fig. 6B). The species demarcation criteria set for the family *Splipalmiviridae* by the ICTV (Sato et al., 2024) are: “members in the same species carry RdRPs share over 70% amino acid sequence identity for both subunits.” If these are adopted, LfNLV1 is regarded as representing a new species.

### 3.3 Utilization of circular or concatemerized forms of viral RNAs for determining the terminal genomic sequences

In this study, we used two methods for determining the terminal sequences of some viral genomic RNAs: RLM-RACE and circular RACE (cirRACE) without RNA circularization steps, the latter being able to amplify RNA regions comprising the tail-to-head linked termini. Unless otherwise mentioned, only conventional RLM-RACE was employed. The methods turned out to be applicable to LfMvV1, SlBoV1, LfNLV1, and LfCV1, and provided near-identical sequences for each terminus in most cases. Occasionally, the terminal sequences obtained by cirRACE were slightly different from those obtained by RLM-RACE, as observed for the dimer S (ambisense) segment molecules of tospoviruses (Bertran et al., 2016). Mapping patterns for the terminally overlapping reads of LfNLV1 RNA3 are shown in Fig. S2. This suggests that in infected mycelia by these viruses accumulate either the viral circular RNA whether covalently linked or not, concatemerized RNA or both. Further confirmatory experiments are needed to determine which type of RNA molecules are accumulated in mycelia infected by LfMvV1, SlBoV1, LfNLV1, and LfCV1. cirRACE is easier than RLM-RACE, as the first requires no adapter ligation to RNA substrates. cirRACE can be used for other mycoviruses such as a partitivirus (H. Kondo and F. Fujimori, unpublished data). Therefore, cirRACE may serve as an alternative to classic RACE and RLM-RACE (Deakin et al., 2017) for some RNA fungal viruses.

The best characterized RNA virus, known to accumulate a circular form of viral RNA (Forgia et al., 2023), is ambiviruses recently classified into a new phylum *Ambiviricota* (Kuhn et al., 2024; Simmonds et al., 2024). Ambiviruses generally encode one or two ribozymes in addition to RdRP and a hypothetical protein, and replicate in a rolling-circle mode. Thus, they are regarded as an intermediate between viroids and RNA viruses (Forgia et al., 2022). In addition to ambiviruses, other negative sense (-)RNA and (+)RNA viruses were shown or suspected to produce a circular form of viral RNA, while not necessarily producing covalently linked circular RNA. Examples include yadokariviruses (Jia et al., 2022), narnaviruses (Matsumoto et al., 1990), mitoviruses (Hintz et al., 2013; Lakshman et al., 1998; Shamsi et al., 2022), and chuviruses (Li et al., 2015). Interestingly, many other RNA mycoviruses such as fusarivirids, hypovirids, fusagravirids, megabirnavirids, orthototivirids, and chrysovirids were experimentally confirmed or bioinformatically predicted to encode ribozymes at the their 5′ terminal or intergenic regions (López-Galiano et al., 2024). The authors speculate a possible role for ribozyme in translation rather than in replication for these viruses with ribozymes. Thus, the relationship between the presence of ribozymes and possible circular or concatemerized viral RNAs is unclear. This study raises interesting open questions such as what form of viral RNAs are accumulated in LfMvV1 and LfCV1-infected fungal colonies? Are circular or concatemerized RNAs are produced in other viruses in addition to those reported in this and previous studies?

## 4 Conclusion

To date very little is known about the mycoviruses of mushroom-pathogenic fungi, contrasting the situation of phytopathogenic fungi, for which hypovirulence- and hypervirulence-conferring viruses are known (Kondo et al., 2022; Wagemans et al., 2022; Xie and Jiang, 2024). The long-term objective of this study is to identify the potential role of mycoviruses in reducing the virulence of *L. fungicola*, as part of an integrated pest management approach to disease control. As a first step, the current study represents the first virome analysis of a collection of 57 fungal isolates (Table S1) associated with mushroom dry bubble disease, i.e., largely *L. fungicola* isolates and minorly *Simplicillum* spp. and *Akanthomyces* sp. While *L. fungicola* has been an established causal agent of mushroom dry bubble disease (Berendsen et al., 2010), the latter two fungal species as possible mushroom pathogens will be reported elsewhere. All of these fungi taxonomically belong to the same family Cordycipitaceae. We determined the complete genomic sequences of a total seven new viral strains belonging to three previously established or proposed species and four novel species. It should be noted that some detected viruses from *L. fungicola* exhibited high levels of sequence identity to those previously reported from an entomopathogenic ascomycete *Beauveria bassiana*, taxonomically classified into the same family Cordycipitaceae as *L. fungicola* (Table 1).

## CRediT authorship contribution statement

**Lóránt Hatvani:** Writing – review & editing, Writing – original draft, Methodology, Investigation, Funding acquisition, Data curation, Conceptualization. **Sakae Hisano:** Writing – review & editing, Visualization, Methodology, Investigation. **Hideki Kondo:** Writing – review & editing, Visualization, Methodology, Investigation, Funding acquisition, Formal analysis. **Hitomi Sugahara:** Writing – review & editing, Visualization, Investigation. **Paul Telengech:** Writing – review & editing, Investigation. **Sabitree Shahi:** Writing – review & editing, Investigation. **Sarah Remi Ibiang:** Writing – review & editing, Investigation. **Sándor Kocsubé:** Writing – review & editing, Resources. **Tünde Kartali:** Writing – review & editing, Resources. **David A. Fitzpatrick:** Writing – review & editing, Supervision. **Helen Grogan:** Writing – review & editing, Writing – original draft, Supervision, Resources, Methodology, Investigation, Conceptualization. **Nobuhiro Suzuki:** Writing – review & editing, Writing – original draft, Visualization, Supervision, Resources, Methodology, Investigation, Funding acquisition, Formal analysis, Data curation, Conceptualization.

## Declaration of competing interest

The authors declare no conflict of interests.

## Data Availability

GenBank accession numbers have been provided.

## Appendix Supplementary materials

Image, application 1

Image, application 2
