# Supplementary material for: Virome of the fungi associated with mushroom dry bubble disease
Source: Virus Res. 2026 Mar 18;367:199714. doi: 10.1016/j.virusres.2026.199714 (PMC13068599; doi:10.1016/j.virusres.2026.199714)
Supplement: Supplementary file 1 [file mmc1.pdf]

Supplementary information for:

Virome of the fungi associated with mushroom dry bubble disease

Lóránt Hatvani[1,2+], Sakae Hisano[2], Hideki Kondo[2], Hitomi Sugahara[2], Paul Telengech[2++], Sabitree Shahi[2+++], Sarah Remi Ibinga[2], Sándor Kocsubé[3], Tünde Kartali[3], David A. Fitzpatrick[4], Helen Grogan[1*], Nobuhiro Suzuki[2,5*]

[1]Teagasc Food Research Centre, Horticulture Development Department; Ashtown, Dublin 15, D15KN3K, Ireland

[2]Institute of Plant Science and Resources, Okayama University; Chuou 2-20-1, Kurashiki, Okayama 710-0046, Japan

[3]Department of Biotechnology and Microbiology, Faculty of Science and Informatics, University of Szeged; Szeged, Közép fasor 52., H-6726, Hungary

[4]Genome Evolution Laboratory, Department of Biology, Maynooth University; Maynooth, Co. Kildare, Ireland.

[5]Neovirology Laboratory, Graduate School of Agricultural Science, Tohoku University; Sendai, Japan.

*Correspondence may be sent to N. Suzuki or H. Grogan

Nobuhiro Suzuki
IPSR, Okayama University
Chuou 2-20-1, Kurashiki, JAPAN
Telephone: 81-86-434-1230
FAX: 81-86-434-1232
E-mail: <nsuzuki@okayama-u.ac.jp>

Helen Grogan
Teagasc Food Research Centre
Horticulture Development Department
Ashtown, Dublin 15, D15KN3K, Ireland
E-mail: <helen.grogan16@gmail.com>

Figure Legend

Fig. S1. Genome organization and phylogeny of Lecanicillium fungicola chrysovirus 1 (LfCV1) and Akanthomyces sp chrysovirus 1 (AsCV1).

(A, B) Schematic diagram of the four genomic dsRNA segments of two alphachrysoviruse LfCV1 and AsCV1. The segment length (in bp) of dsRNA1 to dsRNA4 is shown on the right. The single ORF on each genomic segment, shown by colored boxes, would encode P1 (RNA-dependent RNA polymerase), P2 (capsid protein), P3, or P4, respectively. The map positions of the start/stop codons are denoted on each ORF. The coding strands of the four genomic segments share the terminally conserved sequence stretches, 5'-AUAAAAAACAAAA---GGUUUAAAAGCG-3' for LfCV1 and 5'-AUAAAAAACAAAAAUCC---AAAGCG-3' for AsCV1, respectively. (C) Phylogenetic relationships of LfCV and alphachrysoviruses. The ML tree was constructed based on the MAFFT alignment of alphachrysovirus RdRP sequences, using the LG+F+I+G4 model as the best-fit substitution model. Two members of the genus *Betachrysovirus* were used as outgroups.

Fig. S2. Mapping of reads spanning the terminal junctions of LfNLV1 RNA3.

Raw reads obtained by high-throughput sequencing were mapped to the concatemerized cDNA sequence of Lecanicillium fungicola narna-like virus 1 (LfNLV1) RNA3. Most reads showed the sequence 5'-UUUCA---GGUUUCGGUUUAAACCGAAAAA-3', containing deletions and insertions relative to the terminal sequence 5'-UUUUUUUUCA---GGGUUUCGcUUAAgCGAAAAAA-3' determined by RLM-RACE (Fig. 4).

Fig. S1

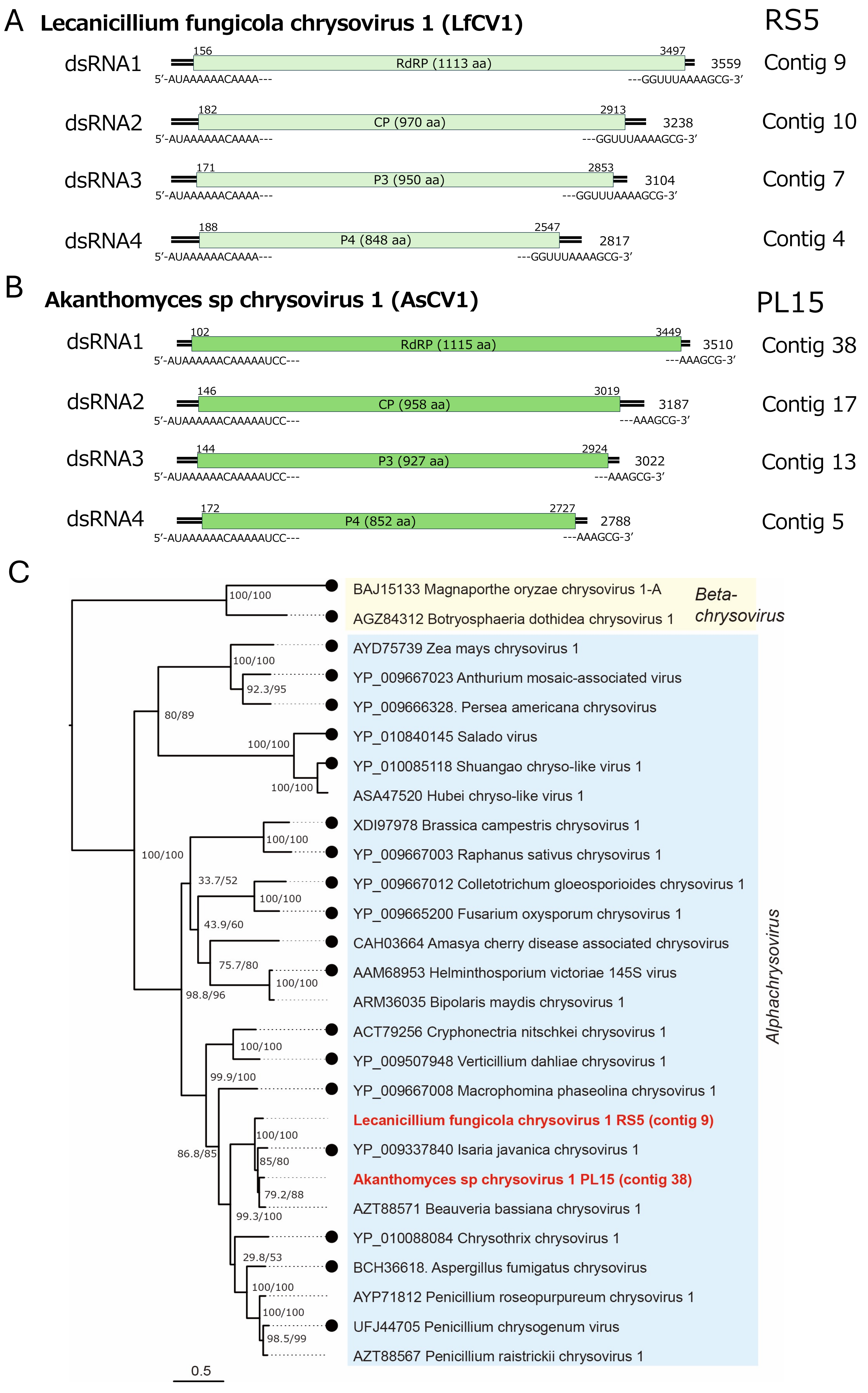
A
Lecanicillium fungicola chrysovirus 1 (LfCV1)
RS5
dsRNA1
156
RdRP (1113 aa)
3497
3559
5′-AUAAAAAAACAAAA---
---GGUUUAAAAGCG-3′
Contig 9
dsRNA2
182
CP (970 aa)
2913
3238
5′-AUAAAAAAACAAAA---
---GGUUUAAAAGCG-3′
Contig 10
dsRNA3
171
P3 (950 aa)
2853
3104
5′-AUAAAAAAACAAAA---
---GGUUUAAAAGCG-3′
Contig 7
dsRNA4
188
P4 (848 aa)
2547
2817
5′-AUAAAAAAACAAAA---
---GGUUUAAAAGCG-3′
Contig 4
B
Akanthomyces sp chrysovirus 1 (AsCV1)
PL15
dsRNA1
102
RdRP (1115 aa)
3449
3510
5′-AUAAAAAAACAAAAAUCC---
---AAAGCG-3′
Contig 38
dsRNA2
146
CP (958 aa)
3019
3187
5′-AUAAAAAAACAAAAAUCC---
---AAAGCG-3′
Contig 17
dsRNA3
144
P3 (927 aa)
2924
3022
5′-AUAAAAAAACAAAAAUCC---
---AAAGCG-3′
Contig 13
dsRNA4
172
P4 (852 aa)
2727
2788
5′-AUAAAAAAACAAAAAUCC---
---AAAGCG-3′
Contig 5
C
100/100
BAJ15133 Magnaporthe oryzae chrysovirus 1-A
AGZ84312 Botryosphaeria dothidea chrysovirus 1
Beta-
chrysovirus
100/100
92.3/95
80/89
100/100
100/100
AYD75739 Zea mays chrysovirus 1
YP_009667023 Anthurium mosaic-associated virus
YP_009666328. Persea americana chrysovirus
YP_010840145 Salado virus
YP_010085118 Shuangao chryso-like virus 1
ASA47520 Hubei chryso-like virus 1
100/100
100/100
33.7/52
100/100
43.9/60
75.7/80
100/100
98.8/96
XDI97978 Brassica campestris chrysovirus 1
YP_009667003 Raphanus sativus chrysovirus 1
YP_009667012 Colletotrichum gloeosporioides chrysovirus 1
YP_009665200 Fusarium oxysporum chrysovirus 1
CAH03664 Amasya cherry disease associated chrysovirus
AAM68953 Helminthosporium victoriae 145S virus
ARM36035 Bipolaris maydis chrysovirus 1
100/100
99.9/100
86.8/85
100/100
85/80
79.2/88
99.3/100
29.8/53
100/100
100/100
98.5/99
ACT79256 Cryphonectria nitschkei chrysovirus 1
YP_009507948 Verticillium dahliae chrysovirus 1
YP_009667008 Macrophomina phaseolina chrysovirus 1
Lecanicillium fungicola chrysovirus 1 RS5 (contig 9)
YP_009337840 Isaria javanica chrysovirus 1
Akanthomyces sp chrysovirus 1 PL15 (contig 38)
AZT88571 Beauveria bassiana chrysovirus 1
YP_010088084 Chrysothrix chrysovirus 1
BCH36618. Aspergillus fumigatus chrysovirus
AYP71812 Penicillium roseopurpureum chrysovirus 1
UFJ44705 Penicillium chrysogenum virus
AZT88567 Penicillium raistrickii chrysovirus 1
Alphachrysovirus
0.5

Fig. S2

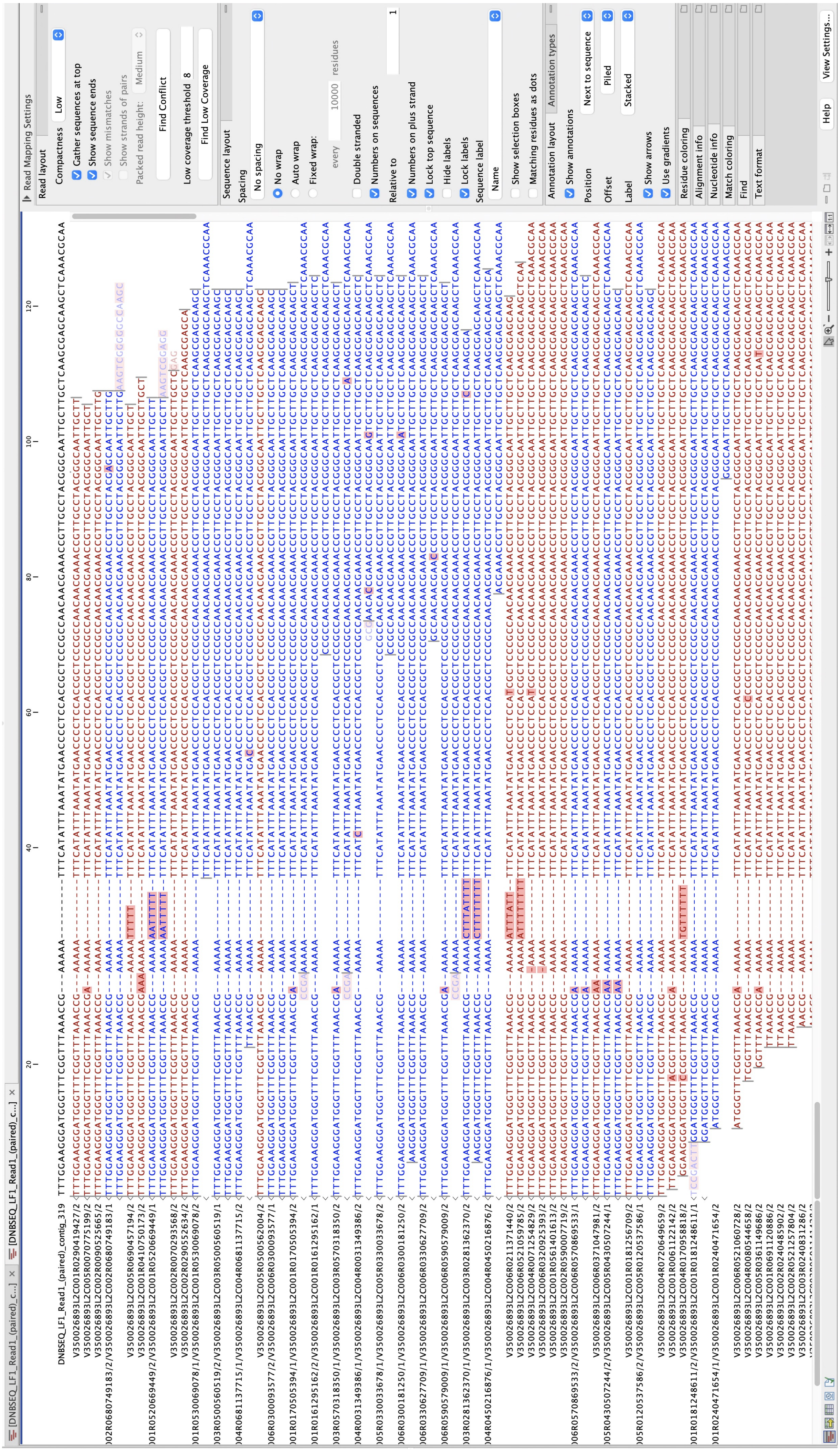

Read Mapping Settings
Read layout
Compactness
Gather sequences at top
Show sequence ends
Show mismatches
Show strands of pairs
Packed read height:
Find Conflict
Low coverage threshold
Find Low Coverage
Sequence layout
Spacing
No spacing
No wrap
Auto wrap
Fixed wrap:
Double stranded
Numbers on sequences
Relative to
Numbers on plus strand
Lock top sequence
Hide labels
Lock labels
Sequence label
Name
Show selection boxes
Matching residues as dots
Annotation layout
Annotation types
Show annotations
Position
Next to sequence
Offset
Piled
Label
Stacked
Show arrows
Use gradients
Residue coloring
Alignment info
Nucleotide info
Match coloring
Find
Text format
Help
View Settings...

Table S1. Data of fungal strains examined during the study

Lab code	Strain ID	Species	Isolation data (Origin, country, year)	Reference/ Source*	HTS analysis (virus detected)	ITS sequence**
**IE1**	**CR181**	***Lecanicillium fungicola* var. *fungicola***	***Agaricus bisporus*, Ireland, 2007**	**This study/A**	**Yes (LfPmV1)**	**PP182280**
IE2	L.2	*Lecanicillium fungicola* var. *fungicola*	*Agaricus bisporus*, Ireland, 2007	This study/B		PP182288
IE3	L.3	*Lecanicillium fungicola* var. *fungicola*	*Agaricus bisporus*, Ireland, 2007	This study/B		PP182292
IE4	L.18	*Lecanicillium fungicola* var. *fungicola*	*Agaricus bisporus*, Ireland, 2007	This study/B		PP182287
IE5	L.20	*Lecanicillium fungicola* var. *fungicola*	*Agaricus bisporus*, Ireland, 2007	This study/B		PP182289
IE6	L.23	*Lecanicillium fungicola* var. *fungicola*	*Agaricus bisporus*, Ireland, 2008	This study/B		PP182290
IE7	L.40	*Lecanicillium fungicola* var. *fungicola*	*Agaricus bisporus*, Ireland, 2008	This study/B		PP182293
IE8	L.43	*Lecanicillium fungicola* var. *fungicola*	*Agaricus bisporus*, Ireland, 2008	This study/B		PP182294
**IE9**	**L.48**	***Lecanicillium fungicola* var. *fungicola***	***Agaricus bisporus*, Ireland, 2009**	**This study/B**	**Yes (LfPmV1)**	**PP182295**
IE10	L.50	*Lecanicillium fungicola* var. *fungicola*	*Agaricus bisporus*, Ireland, 2009	This study/B		PP182296
IE11	L.51	*Lecanicillium fungicola* var. *fungicola*	*Agaricus bisporus*, Ireland, 2009	This study/B		PP182297
IE12	L.52	*Lecanicillium fungicola* var. *fungicola*	*Agaricus bisporus*, Ireland, 2009	This study/B		PP182298
IE13	L.244	*Lecanicillium fungicola* var. *fungicola*	*Agaricus bisporus*, Ireland, 2008	This study/A		PP182291
IE16	1711	*Lecanicillium fungicola* var. *fungicola*	*Agaricus bisporus*, Ireland, 2021	This study/A		PP182284
IE17	1716	*Lecanicillium fungicola* var. *fungicola*	*Agaricus bisporus*, Ireland, 2021	This study/A		PP182285

IE18	1717	*Lecanicillium fungicola* var. *fungicola*	*Agaricus bisporus*, Ireland, 2021	This study/A		PP182286
IE14	1722	*Lecanicillium fungicola* var. *fungicola*	*Agaricus bisporus*, Ireland, 2020	Clarke et al. (2022)/A	Yes	PP182283
IE15	1723	*Lecanicillium fungicola* var. *fungicola*	*Agaricus bisporus*, Ireland, 2020	This study/A		PP182270
HU1	SZMC 25736	*Lecanicillium fungicola* var. *fungicola*	*Agaricus bisporus*, Hungary, 2019	This study/C		PP182302
HU2	SZMC 25737	*Lecanicillium fungicola* var. *fungicola*	*Agaricus bisporus*, Hungary, 2019	This study/C		PP182301
**HU5**	**I3**	***Lecanicillium fungicola* var. *fungicola***	***Agaricus bisporus*, Hungary, 2022**	**This study/D**	**Yes (LfPV1, LfPmV1)**	**PP182282**
**RS1**	**Re4V4**	***Lecanicillium fungicola* var. *fungicola***	***Agaricus bisporus*, Serbia, 2004**	**This study/E**	**Yes (LfCV1)**	**PP182300**
**RS2**	**Zem2V6**	***Lecanicillium fungicola* var. *fungicola***	***Agaricus bisporus*, Serbia, 2006**	**This study/E**	**Yes (LfCV1)**	**PP182307**
RS3	Ša2V6	*Lecanicillium fungicola* var. *fungicola*	*Agaricus bisporus*, Serbia, 2006	Stanojević et al. (2019)/E		PP182303
**RS4**	**BC1V10**	***Lecanicillium fungicola* var. *fungicola***	***Agaricus bisporus*, Serbia, 2010**	**This study/E**	**Yes (LfCV1)**	**PP182277**
**RS5**	**BC3V10**	***Lecanicillium fungicola* var. *fungicola***	***Agaricus bisporus*, Serbia, 2010**	**This study/E**	**Yes (LfMvV1, LfCV1)**	**PP182278**
RS6	P4V2	*Lecanicillium fungicola* var. *fungicola*	*Agaricus bisporus*, Serbia, 2002	This study/E		PP182299
PL1	V25.02	*Lecanicillium fungicola* var. *fungicola*	*Agaricus bisporus*, Poland, 2013	This study/F		PP182305
PL2	V-18	*Akanthomyces* sp.	*Agaricus bisporus*, Poland, 2008	This study/F		PP187028
**PL3**	**S3.7.10**	***Akanthomyces* sp.**	***Agaricus bisporus*, Poland, 2014**	**This study/F**	**Yes (AsCV1)**	**PP187027**
PL4	15A	*Lecanicillium fungicola* var. *fungicola*	*Agaricus bisporus*, Poland, 2008	This study/F		PP182269

PL5	20A	*Lecanicillium fungicola* var. *fungicola*	*Agaricus bisporus*, Poland, 2008	Szumigaj-Tarnowska et al. (2012)/F		PP182271
PL6	25A	*Lecanicillium fungicola* var. *fungicola*	*Agaricus bisporus*, Poland, 2008	This study/F		PP182272
PL7	29A	*Lecanicillium fungicola* var. *fungicola*	*Agaricus bisporus*, Poland, 2008	Szumigaj-Tarnowska et al. (2012)/F		PP182273
PL8	30A	*Lecanicillium fungicola* var. *fungicola*	*Agaricus bisporus*, Poland, 2009	Szumigaj-Tarnowska et al. (2012)/F		PP182274
PL9	V3.7.10	*Akanthomyces* sp.	*Agaricus bisporus*, Poland, 2014	This study/F		PP187026
PL10	V-27	*Lecanicillium fungicola* var. *fungicola*	*Agaricus bisporus*, Poland, 2008	This study/F		PP182304
PL11	V15.06	*Lecanicillium fungicola* var. *fungicola*	*Agaricus bisporus*, Poland, 2013	This study/F		PP182306
PL12	V16A	*Akanthomyces* sp.	*Agaricus bisporus*, Poland, 2008	This study/F		PP187029
**PL13**	**V1X**	***Akanthomyces* sp.**	***Agaricus bisporus*, Poland, 2010**	**This study/F**	**Yes (AsCV1)**	**PP187023**
PL14	V8	*Simplicillum lamellicola*	*Agaricus bisporus*, Poland, 2008	This study/F	Yes (SlBoV1)	PP187205
**PL15**	**4XX**	***Akanthomyces* sp.**	***Agaricus bisporus*, Poland, 2010**	**This study/F**	**Yes (AsCV1)**	**PP187024**
PL16	V20	*Akanthomyces* sp.	*Agaricus bisporus*, Poland, 2008	This study/F		PP187025
UK1	431	*Lecanicillium fungicola* var. *fungicola*	*Agaricus bisporus*, United Kingdom, 1997	This study/A		PP182275
UK2	620	*Lecanicillium fungicola* var. *fungicola*	*Agaricus bisporus*, United Kingdom, 1997	This study/A	Yes	PP182276
CBS 440.34	CBS 440.34	*Lecanicillium fungicola* var. *fungicola*	*Agaricus bisporus*, United Kingdom, 1933	Amey et al. (2002)/G	Yes	PP182279

MAFF 305218	MAFF 305218	*Lecanicillium fungicola* var. *aleophilum*	Japan	This study/H	Yes	https://www.gene.affrc.go.jp/databases-micro_search_detail_en.php?maff=305218
TUFC 65020	TUFC 65020	*Lecanicillium fungicola* var. *aleophilum*	*Agaricus bisporus*, Japan, 1985	This study/I	Yes	https://fungusdb.muses.tottori-u.ac.jp/en/catalog/info?id=65020
NBRC 30728	NBRC 30728/ CBS 300.70A	*Lecanicillium fungicola* var. *aleophilum*	Soil in rain forest, Australia	Gams and Van Zaayen (1982)/J	Yes (LfNLV1)	PP182404
DC 114	DC 114	*Simplicillium aogashimaense*	*Agaricus bisporus*, USA, 1951	Collopy et al. (2001)/K	Yes	PP187094
DC 130	DC 130	*Lecanicillium fungicola* var. *fungicola*	*Agaricus bisporus*, Korea, 1971	Collopy et al. (2001)/K		PP182281
DC 131	DC 131	*Simplicillium aogashimaense*	*Agaricus bisporus*, Switzerland, Early 1970's	Collopy et al. (2001)/K		PP187095
DC 142	DC 142	*Lecanicillium fungicola* var. *aleophilum*	*Agaricus bisporus*, Canada, 1979	Collopy et al. (2001)/K		PP182407
DC 145	DC 145	*Lecanicillium fungicola* var. *aleophilum*	*Agaricus bisporus*, USA, 1979	Collopy et al. (2001)/K		PP182405
DC 150	DC 150	*Lecanicillium fungicola* var. *aleophilum*	*Pleurotus ostreatus*, USA, 1981	Collopy et al. (2001)/K		PP182406
DC 170	DC 170	*Lecanicillium fungicola* var. *aleophilum*	*Agaricus bisporus*, USA, 1982	Potočnik et al. (2008)/K		PP182402
DC 262	DC 262	*Lecanicillium fungicola* var. *aleophilum*	*Agaricus bisporus*, Canada, 1988	Collopy et al. (2001)/K		PP182408

Bold: dsRNA-positive; *A: Teagasc, Ireland; B: National University of Ireland, Maynooth, Ireland; C: Szeged Microbiology Collection, Hungary (http://szmc.hu/); D: Institute of Horticultural Science, Hungary; E: Institute of Pesticides and Environmental Protection, Serbia; F: The National Institute of Horticultural Research, Poland; G: Centraalbureau voor Schimmelcultures, Netherlands (https://wi.knaw.nl/); H: The Research Center of Genetic Resources, National Agriculture and Food Research Organization, Japan (https://www.gene.affrc.go.jp/index_en.php); I: Fungus/Mushroom Resource and Research Center, Japan (https://fungusdb.muses.tottori-u.ac.jp/en/catalog/search); J: NITE

Biological Resource Center, Japan (https://www.nite.go.jp/en/nbrc/cultures/nbrc/index.html); K: The Pennsylvania State University, USA (https://sites.psu.edu/mushroomspawnlab/diseases/); HTS: High-throughput sequencing; **: NCBI GenBank accession number

Table S2. Primer list

Primer Name	Primer Sequence	Purpose	Hosting Strain	Positions		
LfPV1_RdRP-F	GATGTTGAACGGGAAGAGCTC	RT	HU5	822	>	842
LfPV1_RdRP-R	GAAATCTCTGTCCCACATGGC	RT	HU5	1498	<	1518
LfPmV1_RdRP-F	CTGTGAATGAGTCGAAGCTGG	RT	HU5	2009	>	2029
LfPmV1_RdRP-R	GCTAGTTGTTGTACACCACCC	RT	HU5	2306	<	2326
AsCV1_RdRP-F	GCTCGATATGACATGGTGAGAG	RT	PL15	309	>	330
AsCV1_RdRP-R	GAACTTGCCCAGTCTCAAACAAC	RT	PL15	938	<	960
LfMvV1_Mt-F	GTAGCAATCCTCGAGCATGACTG	RT	RS5	851	>	873
LfMvV1_Mt-R	GTAATTCTTGCCAGCATGTCATCC	RT	RS5	1093	<	1116
LfCV1_RdRP-F	GGCCAACACTATCGAGGAGATAC	RT	RS5	1817	>	1839
LfCV1-RdRP-R	CGCCAGCCAGAATACAGACCT	RT	RS5	2309	<	2329
LfNLV1_RdRP-F	CTAGAGTCGCACACCTGATCT	RT	NBRC30728	694	>	714
LfNLV1_RdRP-R	CTCCCTCTTAATGCCAGCTTC	RT	NBRC30728	1197	<	1217
SlBoV1-F	GGTTCCGTGAGCTCTTTTACC	RT	PL14	1481	>	1501
SlBoV1-R	CTTTTAAATACCCGATGCGCCAG	RT	PL14	1910	<	1932
(RACE Adoptor)	PO4-CAATACCTTCTGACCATGCAGTGACAGTCAGCATG	RLM-RACE	All			
RACE 1st	CATGCTGACTGTCACTGCAT	RLM-RACE	All			
RACE 2nd	TGCATGGTCAGAAGGTATTG	RLM-RACE	All			
LfPV1_RNA1-5'R	GTAAGTGTCTCGAGAGTAAGTTTTTAA	RACE	HU5	286	<	312
LfPV1_RNA1-3'F	CGTTAACTCGACTGGTCGGC	RACE	HU5	1463	>	1482
LfPV1_RNA2-5'R	GGTAACACTAGTTCCCCCAGAA	RACE	HU5	268	<	289
LfPV1_RNA2-3'F	TTGACTGCTTGTTTCCCTGTATCT	RACE	HU5	1301	>	1324
LfPmV1_RNA1-5'R	GGCCCTGGTAGTCCTGAAC	RACE	HU5	282	<	300
LfPmV1_RNA1-3'F	GCCAGCCTTCTGCCCCGT	RACE	HU5	2422	>	2128
LfPmV1_RNA2-5'R	TCCATGTCTCGGAGAGTGGC	RACE	HU5	291	<	310
LfPmV1_RNA2-3'F	CGCTCGTTACCCTTGATGCAG	RACE	HU5	1891	>	1911
LfPmV1_RNA3-5'R	ACCGTACTCATACAACTCGAGGT	RACE	HU5	247	<	269
LfPMV1_RNA3-3'F	CCCGGCCATCGAGGGCGA	RACE	HU5	1730	>	1747
LfPmV1_RNA4-5'R	TGGGGAAGGGGAGGAGGCG	RACE	HU5	263	>	281
LfPmV1_RNA4-3'F	CGGTGCACTTCCGAGCGCT	RACE	HU5	882	>	900
AsCV1_RNA1-5'R	CTTTCTCGCGGCTTTAATTGC	RACE	PL15	177	<	197
AsCV1_RNA1-3'F	CATAACTGGCTGGGATGTTG	RACE	PL15	2351	>	2370
AsCV1_RNA2-5'R	CCATCGGCAGACTCAATATG	RACE	PL15	1225	<	1244
AsCV1_RNA2-3'F	GCAGGAATGATGTACGTGATC	RACE	PL15	621	>	640
AsCV1_RNA3-5'R	GTCCATCAATTGCTCACTCC	RACE	PL15	238	<	257
AsCV1_RNA3-3'F	GTTGTGGTGTGCTCCATAC	RACE	PL15	2832	>	2850
AsCV1_RNA4-5'R	GCATCACCAGTACTCACTAG	RACE	PL15	2392	<	2411
AsCV1_RNA4-3'F	GAACATCACCGGATCTATCTG	RACE	PL15	2075	>	2095
LfCV1_RNA1-5'R	AGTCAGTACCTGCCTTAGTCGACC	RACE	RS5	307	<	330
LfCV1_RNA1-3'F	TCAGTCACAAAGCAAGACCTGGTAGAC	RACE	RS5	3261	>	3287
LfCV1_RNA2-5'R	ACCCCCATTGCGTTTCTTGAGCTT	RACE	RS5	209	<	232
LfCV1_RNA2-3'F	GACGTATGCACGACAAGACACTCG	RACE	RS5	2898	>	2921
LfCV1_RNA3-5'R	TGTTTGTGGCTGCTGCCTGTTGTAAA	RACE	RS5	104	>	129
LfCV1_RNA3-3'F	AGACCATCCGGTGTTCGACCTG	RACE	RS5	2900	>	2921
LfCV1_RNA4-5'R	ATCAACGACCCTCTTAACTTCAGACAT	RACE	RS5	188	<	214
LfCV1_RNA4-3'F	GCTGCTGTATTCGAGCCAGGTAC	RACE	RS5	2615	>	2637
LfMvV1_RNA1-5'R	ACGTCGTCGCCTGTGAGGTGG	RACE	RS5	257	<	277
LfMvV1_RNA1-3'F	TTTAGTGCGATTCTTGATACTGCCGAGA	RACE	RS5	1688	>	1715
LfMvV1_RNA2-5'R	CGTCCTTGGTTGCAAACTCAGATTGTT	RACE	RS5	540	<	566
LfMvV1_RNA1-3'F	TTCGGTCAGAGAAATCATCCAGGTTTT	RACE	RS5	1471	>	1497
LfMvV1_RNA3-5'R	ACGTATAGTCCACTCCAAACTCCTGTA	RACE	RS5	115	<	141
LfMvV1_RNA3-3'F	CACATTCGGATTGGGTTTCGGAGC	RACE	RS5	712	>	735
LfMvV1_RNA4-5'R	GGTTGCTTCCGCCAGACTTGC	RACE	RS5	134	<	154
LfMvV1_RNA4-3'F	CTGTAGACCTGTACCAACTCCGTA	RACE	RS5	645	>	668
LfNLV1-RNA1-5'R	CCGGTGGGCAATGTCTTTAG	RACE	NBRC30728	322	<	341
LfNLV1-RNA1-3'F	GCCACAGAGAGCTTTCCAAG	RACE	NBRC30728	1540	>	1550
LfNLV1-RNA2-5'R	GTCTGGCTCTTCTGTTATCTC	RACE	NBRC30728	1241	<	1261
LfNLV1-RNA2-3'F	TCCTTAAGGAACTGAGGGAAAG	RACE	NBRC30728	1391	>	1412
LfNLV1-RNA3-5'R	TTAGCGGGACCCTTCTTCTC	RACE	NBRC30728	223	<	242
LfNLV1-RNA3-3'F	CAATACACTGCCGGGACTTC	RACE	NBRC30728	1061	>	1080
SlBoV1-5'R	AAGACTTCCCCATCCACAGG	RACE	PL14	778	<	797
SlBoV1-3'F	AGGGTTAACTGGGATTTCGGA	RACE	PL14	1828	>	1848
