# Supplementary material for: Virome of the fungi associated with mushroom dry bubble disease
Source: Virus Res. 2026 Mar 18;367:199714. doi: 10.1016/j.virusres.2026.199714 (PMC13068599; doi:10.1016/j.virusres.2026.199714)
Supplement: Supplementary file 2 [file mmc2.docx]

**Fig. S1 Genome organization and phylogeny of Lecanicillium fungicola chrysovirus 1 (LfCV1) and Akanthomyces sp chrysovirus 1 (AsCV1).**

(A, B) Schematic diagram of the four genomic dsRNA segments of two alphachrysoviruse LfCV1 and AsCV1. The segment length (in bp) of dsRNA1 to dsRNA4 is shown on the right. The single ORF on each genomic segment, shown by colored boxes, would encode P1 (RNA-dependent RNA polymerase), P2 (capsid protein), P3, or P4, respectively. The map positions of the start/stop codons are denoted on each ORF. The coding strands of the four genomic segments share the terminally conserved sequence stretches, 5’-AUAAAAAACAAAA---GGUUUAAAAGCG-3’ for LfCV1 and 5’- AUAAAAAACAAAAAUCC---AAAGCG-3’ for AsCV1, respectively. (C) Phylogenetic relationships of LfCV and alphachrysoviruses. The ML tree was constructed based on the MAFFT alignment of alphachrysovirus RdRP sequences, using the LG+F+I+G4 model as the best-fit substitution model. Two members of the genus *Betachrysovirus* were used as outgroups.
